# The partitioned LASSO-patternsearch algorithm with application to gene expression data

**DOI:** 10.1186/1471-2105-13-98

**Published:** 2012-05-15

**Authors:** Weiliang Shi, Grace Wahba, Rafael A Irizarry, Hector Corrada Bravo, Stephen J Wright

**Affiliations:** 1 Sanofi–Aventis, Cambridge, Massachusetts, USA; 2 Department of Statistics, University of Wisconsin-Madison, Madison, Wisconsin, USA; 3 Department of Biostatistics, Johns Hopkins University, Baltimore, Maryland, USA; 4 Center for Bioinformatics and Computational Biology, Computer Science Department, University of Maryland-College Park, College Park, Maryland, USA; 5 Department of Computer Sciences, University of Wisconsin-Madison, Madison, Wisconsin, USA

## Abstract

**Background:**

In systems biology, the task of reverse engineering gene pathways from data has been limited not just by the curse of dimensionality (the interaction space is huge) but also by systematic error in the data. The gene expression barcode reduces spurious association driven by batch effects and probe effects. The binary nature of the resulting expression calls lends itself perfectly to modern regularization approaches that thrive in high-dimensional settings.

**Results:**

The Partitioned LASSO-Patternsearch algorithm is proposed to identify patterns of multiple dichotomous risk factors for outcomes of interest in genomic studies. A partitioning scheme is used to identify promising patterns by solving many LASSO-Patternsearch subproblems in parallel. All variables that survive this stage proceed to an aggregation stage where the most significant patterns are identified by solving a reduced LASSO-Patternsearch problem in just these variables. This approach was applied to genetic data sets with expression levels dichotomized by gene expression bar code. Most of the genes and second-order interactions thus selected and are known to be related to the outcomes.

**Conclusions:**

We demonstrate with simulations and data analyses that the proposed method not only selects variables and patterns more accurately, but also provides smaller models with better prediction accuracy, in comparison to several alternative methodologies.

## Background

The LASSO-Patternsearch (LPS) algorithm [1-3] is an effective approach for identifying multiple dichotomous risk factors for outcomes of interest in demographic and genomic studies. It uses an *ℓ*_1_-regularized logistic regression formulation, targeting the case in which only a small fraction of the large number of possible candidate patterns are significant. The approach can be used to consider simultaneously all possible patterns up to a specified order. It can identify complicated correlation structures among the predictor variables, on a scale that can cause serious difficulties for algorithms that target problems of more modest size.

When applied to very large models with higher-order interactions between the predictor variables, however, LPS quickly runs into computational limitations. For example, a problem with two thousand predictor variables yields a logistic-regression formulation with about two million variables if both first- and second-order patterns are included in the model. Problems of this size are at the limit of LPS capabilities, yet current problems of interest in genetic epidemiology consider ten thousand markers or more [4]. Data sets of this scale can be addressed by using a screening procedure in conjunction with LPS [5].

In this article, we propose a Partitioned LASSO-Patternsearch Algorithm (pLPS) scheme to tackle gigantic data sets in which we wish to consider second- and possibly third-order interactions among the predictors, in addition to the first-order effects. As in LPS, we assume that all predictor variables are binary (or that they have been dichotomized before the analysis). The model thus contains a huge number of possible patterns, but the solution is believed to be sparse, with only a few effects being significant risk factors for the given outcome. In the first (screening) stage of pLPS, the predictors are divided into partitions of approximately equal size, and LPS is used to solve smaller subproblems in which just the predictors and higher-order effects within a single partition, or the interactions between variables in small groups of partitions, are considered as variables in the optimization model. These reduced problems can be solved independently and simultaneously on a parallel computer. By the end of the screening stage, each predictor and each higher-order effect (up to the specified order) will have been considered in at least one of the subproblems. The second stage of pLPS is an aggregation process, in which all predictors identified in the first stage are considered, together with all their interactions up to the specified order. An LPS procedure is used to identify the final set of significant predictors and interactions.

Tuning parameters in the first stage of pLPS are chosen by BGACV criterion. BGACV is a more stringent criterion than GACV, the difference between these criteria being similar to the difference between BIC and AIC (see [1]). In the second stage, two tuning parameters are used, one for main effects and one for interactions. These are chosen by BGACV2, a variation of BGACV to be described below. We examine the effectiveness of the pLPS strategy on simulated data and on two large-scale genetic data sets.

## Methods

We now give further details of the pLPS scheme and its implementation. For simplicity, most of our discussion focuses on the case in which first-order effects and second-order interactions between all predictors are considered. Extension of the approach to include third-order effects as well is described briefly at the end of the section.

Considering *n* subjects with *p* binary predictor variables, the total number of interactions up to order *q* is given by NB=∑ν=0qpν. For *q *= 2, we thus have 1 + *p*(*p* + 1)/2 patterns. To apply pLPS, we first divide the *p* variables into *k* partitions so that each partition has *g *=* p*/*k*variables. (For simplicity of description, we assume that *p* is divisible by *k*.) The data set is {*y*,*x*_*j*_,*j *= 1,2,⋯ ,*p*}, where *y *= (*y*_1_,*y*_2_,⋯,*y*_*n*_)∈{0,1} is the response, *x*_*j *_= (*x*_*j*_(1),*x*_*j*_(2),⋯ ,*x*_*j*_(*n*)) is the *j*th covariate, and *x*_*j*_(*i*)∈{0,1} for all *j *= 1,2,⋯ ,*p* and *i *= 1,2,⋯ ,*n*. By relabelling the *p* predictors as *x*_*st*_, where *s *= 1,2,⋯ ,*k* denotes the partition number and *t *= 1,2,⋯ ,*g*denotes the index within the partition, we relabel the full data set as {*y*,*x*_*st*_, *s *= 1,2,⋯ ,*k*, *t *= 1,2,⋯ ,*g*}.

In the first stage of pLPS (the “screening stage”), we solve two types of reduced LPS subproblems. The first type is based on a pair of partitions, denoted by *s*_1_and *s*_2_, and defines the LPS variables in the subproblems to be the first-order effects within each group (for which there are *2g* basis functions $\left\{B_{t_{1}}=x_{s_{1}t},\;t=1,2,\cdots \;,g\right\}$ and $\left\{B_{t_{2}}=x_{s_{2}t},\;t=1,2,\cdots \;,g\right\}$) and all the second-order interactions between a predictor in group *s*_1_and a predictor in group *s*_2_. There are *g*^2^ basis functions for the latter effects, namely, $\left\{B_{t_{1}t_{2}}=x_{s_{1}t_{1}}\times x_{s_{2}t_{2}},\;t_{1},t_{2}=1,2,\cdots \;,g\right\}$. Hence the total number of patterns in the LPS model for each subproblem is *g*^2^ + 2*g* + 1, when we include the constant basis function *B *≡ 1.

**Figure 1:**
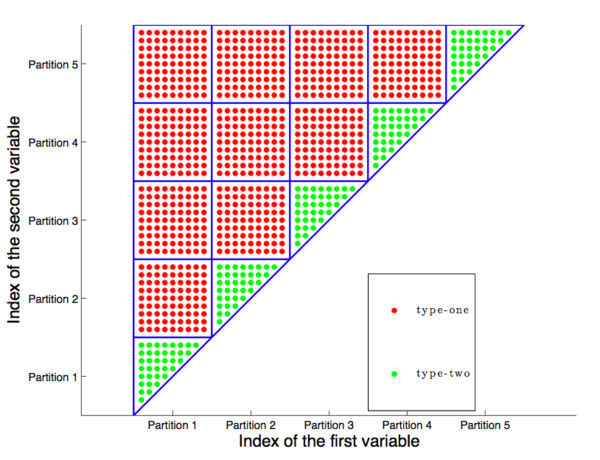
**Diagram of the subproblems in the first stage of pLPS, assuming 5 partitions.** Side length of a square is the partition size, while the horizontal axis contains the labels of the first effect and the vertical axis the label of the second effect. Squares filled with red dots are “type-one” subproblems while the triangles filled with green dots are “type-two” subproblems.

The second type of reduced LPS problem is obtained from the first- and second-order effects within a single partition. Here, the basis functions for group *s* are $\left\{B_{t_{1}t_{2}}=x_{st_{1}}\times x_{st_{2}},\;t_{1},t_{2}=1,2,\cdots \;,g,\;t_{1}<t_{2}\right\}$ and {*B*_*t *_=* x*_*st*_, *t *= 1,2,⋯ ,*g*}, making a total of 1 + *g*(*g* + 1)/2 patterns, when we include the constant basis function. Since each subproblem of the second type has about half as many variables as each subproblem of the first type, we define computational tasks of roughly equivalent complexity by grouping two of the type-two problems together. Figure 1 is a graphical presentation of the two types of groups considered in the first stage of pLPS.

We now briefly describe the LPS methodology, which is applied to each of these subproblems. By relabeling, we define the basis functions to be *B*_*ℓ*_(*x*), *ℓ *= 1,2,⋯ ,*N*_*B*_. Defining $p(x):=\text{Prob}\left[y=1|x\right]$ and the logit (log odds ratio) $f(x):=\;\text{log}\;\left[p(x)/(1-p(x))\right]$, we estimate *f * by minimizing

(1) $$I_{\lambda }(y,f)=ℒ(y,f)+\lambda J(f),$$

where ℒ(y,f) is the negative log likelihood divided by *n*:

(2) $$ℒ(y,f)=\frac{1}{n}\overset{n}{\underset{i=1}{\sum }}\left[-y_{i}f(x(i))+\text{log}\left(1+e^{f(x(i))}\right)\right],$$

with *f * being expressed as a linear combination of the basis functions

(3) $$f(x)=\mu +\overset{N_{B}-1}{\underset{\ell =1}{\sum }}c_{\ell }B_{\ell }(x),$$

and the penalty function being defined by

(4) $$J(f)=\overset{N_{B}-1}{\underset{\ell =1}{\sum }}|c_{\ell }|.$$

(We assume that the last basis function is the constant function 1, whose coefficient *μ *does not appear in *J* and is therefore not penalized.) The penalty parameter *λ* in (1) is chosen by BGACV. We then build a parametric logistic regression model on the remaining basis functions by minimizing (2) and selecting the best model via backward elimination with the BGACV criteria. More details are given in Section Results and discussion [1].

If the outcomes can be predicted well using a small number of patterns, the number of patterns surviving the first stage of pLPS should be small. Suppose there are a total of *p*^∗^ unique predictor variables in all these patterns. The second stage of pLPS — the “aggregation stage” — is an LPS problem in which just these predictors and all their second-order effects are the patterns. There will be $N_{B_{1}}=p^{*}$ basis function (denoted by *B*_1*ℓ*_) for the main effects and $N_{B_{2}}\;(=\left(\begin{array}{c} p^{*}\\ \;2 \end{array}\right))$ basis functions (denoted by *B*_2*ℓ*_) for the second-order interactions, plus one constant basis function. In the aggregation stage, we use different penalty parameters for the first- and second-order patterns, so the objective function is

(5) $$I_{\lambda_{1},\lambda_{2}}(y,f)=ℒ(y,f)+\lambda_{1}J_{1}(f)+\lambda_{2}J_{2}(f),$$

where the link function *f * is

(6) $$f(x)=\mu +\overset{N_{B_{1}}}{\underset{\ell =1}{\sum }}c_{1\ell }B_{1\ell }(x)+\overset{N_{B_{2}}}{\underset{\ell =1}{\sum }}c_{2\ell }B_{2\ell }(x),$$

and the penalties are

(7) $$J_{1}(f)=\overset{N_{B_{1}}}{\underset{\ell =1}{\sum }}|c_{1\ell }|,\;J_{2}(f)=\overset{N_{B_{2}}}{\underset{\ell =1}{\sum }}|c_{2\ell }|.$$

The choice of penalty parameters (*λ*_1_*λ*_2_) in (5) is critical to the performance of this formulation. BGACV does not work well in this setting. Often, it tends to select only second-order patterns, combining main effects with spurious partners. Occasionally, it selects only main effects, breaking true size-two patterns into separate main effects. The large difference between the numbers of basis functions $N_{B_{1}}$ and $N_{B_{2}}$ makes the solutions sensitive to the two penalty parameters. Searching over a grid of values for *λ*_1_ and *λ*_2_ is expensive and often does not give satisfactory results. As an alternative approach, we introduce the following penalty function, known as BGACV2:

(8) $$\begin{array}{l} \text{BGACV}2(\lambda_{1},\lambda_{2})=\text{BGACV}(\lambda_{1},\lambda_{2})\\ \;\;\;\;\times \left(1+0.5\frac{\left|n_{b_{1}}-n_{b_{2}}\right|}{n_{b_{1}}+n_{b_{2}}}\right), \end{array}$$

where $N_{B_{1}}$ is the number of nonzero coefficients of main effects and $N_{B_{2}}$ is the number of nonzero coefficients of size-two patterns. The additional penalty factor forces these two numbers to be similar, reducing the possibility of the two extreme cases discussed above. If the true model only contains main effects, the BGACV2 penalty will tend to select fewer main effects than the BGACV model. However, BGACV is conservative (see discussion in [1]), while BGACV2 is less so. We expect that BGACV2 will not miss any important main effects, though it may also produce some spurious second-order effects. These spurious effects will be further eliminated by the parametric logistic regression step as noted above, followed by solving (5).

Minor extensions to the pLPS approach are needed when size-three patterns (*q *= 3) are introduced. In the screening phase of pLPS, there are four types of subproblems (rather than two). These types are distinguished by considering the labels *s*_1_,*s*_2_,*s*_3_ of the three partitions chosen to define the subproblem (with *s*_1_ ≤* s*_2_ ≤* s*_3_). The four types correspond to the cases *s*_1_ <* s*_2_ <* s*_3_, *s*_1_ =* s*_2_ <* s*_3_, *s*_1_ <* s*_2_ =* s*_3_, and *s*_1_ =* s*_2_ =* s*_3_, respectively. In the aggregation phase of pLPS, we will still be using two penalty parameters, one for main effects and one for interactions; size-two and size-three patterns share the same penalty parameter. The criterion function for choosing the appropriate values for penalty parameters *λ*_1_ and *λ*_2_ is

(9) $$\begin{array}{l} \text{BGACV}3(\lambda_{1},\lambda_{2})=\text{BGACV}(\lambda_{1},\lambda_{2})\\ \;\;\;\;\times \left(1+0.5\frac{\left|n_{b_{1}}-n_{a}|+|n_{b_{2}}-n_{a}|+|n_{b_{3}}-n_{a}\right|}{n_{b_{1}}+n_{b_{2}}+n_{b_{3}}}\right), \end{array}$$

where $N_{B_{1}}$ is the number of nonzero main effects, $N_{B_{2}}$ is the number of nonzero size two patterns, $n_{b_{3}}$ is the number of nonzero size three patterns and *n*_*a *_is the average of the three.

In the remainder of the paper, we use pLPS to denote the *q *= 2 case and pLPS3 for the *q *= 3 case.

The choice of *g* (the number of variables in each partition) is determined by the computing power and the available memory. On our super server (an AMD Dual-Core 2.8 GHz machine with 64 GB memory), we usually set *g *= 2,000 for *q *= 2. This choice yields subproblems with *N*_*B *_= 2,001,001 basis functions, which can be handled comfortably by the LPS code. On a more standard computer (Intel^®^ Pentium^®^ 4 2.80GHz with 2 GB memory), we usually set *g *= 200 for *q *= 2 and *g *= 35 for *q *= 3. As we noted earlier, the subproblems in the first stage of pLPS can be solved independently, in parallel, on different computers in a cluster. The grid-computing system Condor ( http://www.cs.wisc.edu/condor/) provides an ideal platform for these parallel jobs. In our Condor implementation, we request machines from the pool with at least 2 GB of memory, and define our group sizes to be *g *= 200 (for *q *= 2) and *g *= 35 (for *q*=3). Generally, for faster execution of pLPS, it is advantageous to set *g* to the highest value that can be accommodated by the memory of the computer. The final results of the computation do not depend strongly on the choice of *g*.

The pLPS code is available at http://pages.cs.wisc.edu/~swright/LPS/index-plps.html.

## Results and discussion

### Simulation studies

In this section we study the empirical performance of pLPS through four simulated examples. The first example is a relatively small data set with independent predictor variables: One main effect and two second-order interactions are included in the link function. The second example is a very large data set with strong correlations among neighboring variables, in which two main effects and two second-order interactions are assumed to be important. The third example studies the performance of pLPS3, which includes third-order interactions in the model. Two main effects, one second-order interaction, and one third-order interaction are included. The last example studies the performance of pLPS where there are negative correlations among the predictor variables and the true model involves many patterns.

We compare pLPS with three other methods: 

• Logic Regression [6], as implemented in the R package LogicReg

• Stepwise Penalized Logistic Regression (SPLR) [7], as implemented in the R package stepPlr, and

• Random Forest (RF) [8], as implemented in the R package randomForest.

The number of trees and number of leaves in Logic Regression are selected by five-fold cross validation. The smoothing parameter in SPLR is also selected by five-fold cross validation, while the model size is selected by BIC.

#### Simulation Example 1

In our first example, 400 iid Bernoulli (0.5) random variables were simulated. The sample size is 700 and the logit function is

$$f(x)=-2+1.5X_{50}+1.5X_{150}X_{250}+1.5X_{251}X_{252}.$$

One hundred data sets were generated according to this model and analyzed by the four methods described above.

Table 1 presents the results of this simulation. Each entry in the table shows the number of appearances of the pattern and the variables in the 100 simulations. The main number (outside the parentheses) is the pattern count showing how many times the given pattern is selected in the model. The numbers inside the parentheses are the variable counts showing how many times each variable in a given pattern appears in the model, either as a main effect or in some other interaction. Random Forest does not generate an explicit model, but rather produces an importance score for all variables. Thus, it is not possible to calculate a pattern count for Random Forest, but we calculate the variable count according to whether the variables in question appeared among the top 10 variables identified by this technique. For pLPS, Logic Regression, and SPLR, the last column labeled “False Patterns (Variables)” counts the total number of appearances in the 100 trials by terms that are not patterns in the model. In this simulation, any pattern other than *X*_50_, *X*_150_*X*_250_, or *X*_251_*X*_252_ is taken to be false. For Random Forest, the last column counts the total number of false variables selected in the 100 trials. Any variable other than the five in the logit function is false.

**Table 1:** Simulation Example 1

				**False patterns**
**Methods**	***X***_**50**_	***X***_**150**_***X***_**250**_	***X***_**251**_***X***_**252**_	**(Variables)**
pLPS	94 (100)	99 (99,99)	96 (97,97)	153
Logic	100 (100)	70 (88,91)	65 (84,90)	190
RF	NA (100)	NA (96,97)	NA (94,96)	(517)
SPLR	100 (100)	97 (100,97)	91 (100,98)	712

*n *= 700 and *p *= 400, with no correlations. Tabulated numbers show the number of tests (out of 100) in which the pattern was detected by each algorithm. The number outside the parentheses is the number of times the given pattern was selected; the numbers inside the parentheses shows how many times the variables in the pattern are detected in the model, as a main effect or in some interaction. The final column shows the total number of times (in 100 tests) that the algorithms selected patterns (variables for RF) that are not in the true model.

On this example, pLPS selects all three patterns almost perfectly and generates the least number of false patterns. Logic Regression does not do well on the size-two patterns and selects slightly more false patterns. Random Forest does well in selecting the important variables but also selects many false variables. (If we change the criterion for declaring that Random Forest has selected a variable to the “top eight” or “top five,” we reduce the number of false variables but also reduce the variable counts.) SPLR has similar performance to pLPS in selecting the true patterns, but selects many more false patterns.

#### Simulation Example 2

Example 2 studies the behavior of pLPS on a large data set (*n *= 1000, *p *= 8000) with correlations among the covariates. To generate the binary variables *X*_*i*_, *i *= 1,2,⋯ ,*p*, we start with normal distributions, choosing $X_{i}^{*}∼N(0,1)$, *i *= 1,2,⋯ ,*p* so that corr $(X_{i}^{*},X_{i+1}^{*})=2/3$ and corr $(X_{i}^{*},X_{i+2}^{*})=1/3$, *i *= 1,2,⋯ ,*p *− 2. ( $X_{i}^{*}$ and $X_{j}^{*}$ are independent if |*i*−*j*|>2.) We then set *X*_*i *_= 1 if $X_{i}^{*}>0$ and *X*_*i *_= 0 otherwise, for each *i *= 1,2,⋯ ,*p*. The logit function is

$$\;f(x)=-4+2X_{500}+3X_{5000}+2X_{1000}X_{3000}+3X_{7000}X_{7002}.$$

The simulation was performed 50 times; each run is quite time-consuming. We could not run Logic Regression on this example, as the dimensions exceed the limit of that code.

Table 2 shows the results, in the same format as Table 1. pLPS misses the pattern *X*_1000_*X*_3000_ twice but selects the remaining patterns perfectly, and generates a smaller number of false patterns than the other methods. In Random Forest, we declared a variable to be selected if it was ranked in the top 12. It misses the pattern *X*_1000_*X*_3000_ with some frequency. SPLR selects all four patterns perfectly, but at the cost of a large number of spurious patterns. SPLR requires the user to set the maximum number of parameters allowed in the model, and selects the actual number by BIC. We set this maximum to 20, and it was reached on all 50 runs. (The maximum is still reached on every run when we set this parameter to 50).

**Table 2:** Simulation Example 2

					**False patterns**
**Methods**	***X***_**500**_	***X***_**5000**_	***X***_**1000**_***X***_**3000**_	***X***_**7000**_*X*_**7002**_	**(Variables)**
pLPS	50 (50)	50 (50)	48 (48,50)	50 (50,50)	278
RF	NA (50)	NA (50)	NA (28,37)	NA (50,50)	(335)
SPLR	50 (50)	50 (50)	50 (50,50)	50 (50,50)	800

*n *= 1000 and *p *= 8000, with correlations among neighboring variables. Tabulated numbers show the number of tests (out of 50) in which the pattern was detected by each algorithm. The number outside the parentheses is the number of times the given pattern was selected; the numbers inside the parentheses shows how many times the variables in the pattern are detected in the model, as a main effect or in some interaction. The final column shows the total number of times (in 50 tests) that the algorithms selected patterns (variables for RF) that are not in the true model.

#### Simulation Example 3

Example 3 studies the behavior of pLPS3 on a large data set, with sample size *n *= 1000 and *p *= 500 variables. The marginal distribution and correlation structure are the same as in Example 2.

The logit function is

$$\;f(x)=-4+2X_{100}+3X_{200}+2X_{300}X_{400}+3X_{150}X_{450}X_{451}.$$

This simulation was performed 50 times. As we can see from Table 3, pLPS3 selects all patterns quite well with a reasonable number of false patterns. Logic Regression selects fewer false patterns but does not do well in identifying the two interaction terms. Random Forest does well in the size-three pattern but misses the size two pattern quite often. (We declared the top 12 variables identified by Random Forest to be “selected”).

**Table 3:** Simulation Example 3

					**False patterns**
**Methods**	***X***_**100**_	***X***_**200**_	***X***_**300**_***X***_**400**_	***X***_**150**_***X***_**450**_***X***_**451**_	**(Variables)**
pLPS3	47 (50)	50 (50)	47 (50,50)	47 (50,49,48)	204
Logic	50 (50)	50 (50)	34 (43,44)	30 (50,44,41)	151
RF	NA (50)	NA (50)	NA (36,40)	NA (49,47,49)	(279)
SPLR	50 (50)	50 (50)	45 (49,50)	50 (50,50,50)	554

*n *= 1000 and *p *= 500, with correlations among neighboring variables. Tabulated numbers show the number of tests (out of 50) in which the pattern was detected by each algorithm. The number outside the parentheses is the number of times the given pattern was selected; the numbers inside the parentheses shows how many times the variables in the pattern are detected in the model, as a main effect or in some interaction. The final column shows the total number of times (in 50 tests) that the algorithms selected patterns (variables for RF) that are not in the true model.

As in the previous examples, SPLR does well at selecting the important patterns but also selects many false patterns.

#### Simulation Example 4

Simulation 4 studies the performance of pLPS when there are some negative correlations among the covariates and the number of true patterns is large. Assuming *n *= 700 and *p *= 400, the correlation structure of the first 200 variables are the same as those in Example 2. The next 200 variables have some negative correlations generated as follows. Similar to previous examples we start with normal variables, choosing $X_{i}^{*}∼N(0,1)$, *i *= 201,202,⋯ ,*p* so that corr $(X_{i}^{*},X_{i+1}^{*})=-1/3$ and corr $(X_{i}^{*},X_{i+2}^{*})=-1/6$, *i *= 201,202,⋯ ,*p *− 2. ( $X_{i}^{*}$ and $X_{j}^{*}$ are independent if |*i*−*j*|>2.) We then set *X*_*i *_= 1 if $X_{i}^{*}>0$ and *X*_*i *_= 0 otherwise, for each *i *= 201,202,⋯ ,*p*. The logit function is

$$\begin{array}{l} \;f(x)=-1+2.5(X_{1}-X_{3}+X_{10}-X_{201}+X_{220}-X_{230})\\ \;\;+3(X_{100}X_{102}-X_{300}X_{302}+X_{50}X_{250}). \end{array}$$

Among the three interaction terms, the first had variables with positive correlation, the second had variables with negative correlation and the third had independent variables. The simulation was performed 100 times. (Logic Regression was again not implemented. Although the dimensions did not exceed the limit of that code, the number of true patterns did.) Table 4 shows the results. Instead of listing the appearance frequency of all main effects, the average of the seven is presented. pLPS selected all main effects and interactions almost perfectly. In Random Forest, we declared the top 25 variables to be “selected,” but this technique did not do well in identifying the second interaction term. SPLR selected all seven main effects perfectly, but did not do well in selecting the second interaction term, and tended to select many more false patterns.

**Table 4:** Simulation Example 4

					**False patterns**
**Methods**	**Main effects average**^**∗**^	***X***_**100**_***X***_**102**_	***X***_**300**_***X***_**302**_	***X***_**50**_***X***_**250**_	**(Variables)**
pLPS	96 (100)	98 (100,100)	98 (98,100)	99 (100,100)	320
RF	NA (99)	NA (96,100)	NA (87,72)	NA (94,89)	(1268)
SPLR	100(100)	97 (100,100)	82 (100,100)	97 (100,100)	1017

*n *= 700 and *p *= 200, with positive correlations among neighbors in the first 200 variables and negative correlations among neighbors in the next 200 variables. Tabulated numbers show the number of tests (out of 100) in which the pattern was detected by each algorithm. The number outside the parentheses is the number of times the given pattern was selected; the numbers inside the parentheses shows how many times the variables in the pattern are detected in the model, as a main effect or in some interaction. The final column shows the total number of times (in 100 tests) that the algorithms selected patterns (variables for RF) that are not in the true model.

^∗^The average of *X*_1_, *X*_3_, *X*_10_, *X*_201_, *X*_210_, *X*_220_ and *X*_230_.

#### Summary

Logic Regression cannot handle very large data sets and does not reliably identify the interaction terms. Random Forest does not provide an explicit model of the interactions. It frequently scores well, but can perform poorly if the signal is not strong enough. SPLR scores well at selecting the right patterns, but selects too many false patterns. By contrast, pLPS usually selects the right patterns without adding too many false patterns, regardless of the size of the problem, the number of true patterns or the signs of correlations.

Our partitions are selected according to the natural order of variables in these simulation examples. If the number of variables in each partition is 200, the first 200 variables will be in the first partition and the next 200 variables will be in the second partition, and so on. If the variables are permuted, resulting in a different partitioning, we do not expect the results to be greatly affected. All possible higher-order patterns are considered in the first (screening) stage of the method, regardless of partitioning. A significant effect should survive the first stage regardless of how the partitioning is performed. To verify this claim, we performed a random permutation on the predictor variables in simulation Example 4 in all 100 runs. Among all the patterns selected in the original partitioning, 82% were still selected after the permutation. Although this figure is on the low side, it can be accounted for by the presence of noise patterns. If we focus on the ten most important patterns in each run, then from the 1000 considerations (10 patterns x 100 runs), the original partition and its permuted counterpart yield results that agree 95% of the time. To summarize: Although Simulation Example 4 is a complicated case with negative correlations and many important variables, the final results are not affected greatly by a shuffling of the first-stage partitions. We would expect similar results for the other examples discussed in this article.

### The gene expression barcode data

With current microarray technology, we are able to measure thousands of RNA transcripts at one time, a capability that allows for richer characterization of cells and tissues. However, feature characteristics such as probe sequence can cause the observed intensity to be far away from the actual expression. Although this “probe effect” is significant, it is consistent across different hybridizations, in that the effect is quite similar when comparing intensities of different hybridizations for the same gene. Therefore, the majority of microarray data analysis uses relative expression rather than absolute expression. To overcome this limitation in measurement, a gene expression bar code (GEBC) [9] was proposed recently. The goal is to investigate what intensity measurement constitutes “no expression” for a given gene and microarray platform. GEBC starts by preprocessing all genes using Robust Multi-array Analysis (RMA) [10]. For each gene, an empirical density smoother is used to estimate the density function of this gene across tissues, and the smallest mode of the density function is taken to be the expected intensity of an unexpressed gene. Gene expressions to the left of this mode are used to estimate the standard deviation of unexpressed genes. If the log expression estimate of a gene is some constant *K* standard deviations larger than the unexpressed mean, then this gene is considered to be expressed. (*K* is chosen to be 6 by cross-validation.) For the purpose of our model, expressed genes are coded as 1 and unexpressed genes as 0.

GEBC [9] was built from publicly available raw data from 40 different studies. It consists of a database of 1094 human samples, representing 118 different tissues. Of these samples, 503 are normal, 500 are breast tumors, and 91 are other diseases. A total of 22,215 genes are available for each sample. We dichotomize these genes in the manner described above. Genes that are expressed in fewer than 10% or more than 90% of the tissues are removed from our analysis; 7,654 genes remained after this screening step. (Genes with unbalanced expression levels, i.e., with very low or high expression rate, are generally not helpful in prediction.) As discussed earlier, there is essentially no limit on the number of variables that can be analyzed by pLPS, but reducing the problem size from 22,215 to 7,654 saves significant computation time.

In our first analysis, we took all normal tissues as “controls” and all non-breast tumor tissues as “cases”. In the second analysis we analyze the survival time of breast cancer patients after dichotomization. We define subjects with survival time less than 5 years as “cases” and those with survival time longer than 10 years as “controls”.

We apply pLPS on both data sets with 7,654 genes, evaluating the variable selection performance of pLPS by comparing with the knowledge base in literature. To compare the performance of pLPS with the alternative methods discussed in the Simulation section, the number of predictor genes must be reduced further, because Logic Regression cannot handle more than 1,000 variables. A screen step [5] was implemented to perform the required reduction. In this step, we fitted a simple logistic regression on each gene and selected the most significant genes based on the p-values from the regression models.

#### Non-Breast Cancer data

In this analysis, all normal and non-breast cancer tissues are used. Breast tumors were excluded because no normal breast tissue was available. The data set contains 503 normal tissues and 70 cancer tissues, giving a malignancy rate of 12.2*%*.

The model fitted by pLPS on this data with 7,654 genes is shown in (10). Five size-two interactions are selected.

(10) $$\begin{array}{l} \;f=-8.15+3.58\times \text{CALU}\times \text{ERBB}3+1.93\;\times \;\text{LAMC}1\\ \;\times \text{CD}24+3.29\times \text{LPCAT}1\times \text{ACY}1+3.75\\ \;\times \text{FXYD}3\times \text{GNL}3+2.34\times \text{NOTCH}3\times \text{CD}24. \end{array}$$

Most of these genes are known to be related to one or more types of cancer. For example, ERBB3 is very important in the development of breast cancer [11] and prostate cancer [12]. LPCAT1 is shown to be highly overexpressed in colorectal adenocarcinomas, when compared to normal mucosas [13]. ACY1 is found to be underexpressed in small-cell lung cancer (SCLC) cell lines and tumors [14]. FXYD3 is overexpressed in pancreatic ductal adenocarcinoma and influences pancreatic cancer cell growth [15]. Notch3 overexpression is common in pancreatic cancer [16]. Finally, CD24, one of the most well-known genes in this model, is related to breast cancer, ovarian cancer, NSCLC, and colorectal cancer [17-20].

To reduce the number of predictor genes to the size that is solvable by alternative methods, we fitted a simple logistic regression on each gene and kept the most significant genes (p-value <10^−8^). This step yields 636 genes. Although this screening step results in the loss of many genes that could potentially be helpful in prediction, it must be performed in order to apply the alternative methods. To yield a fair comparison, we run all methods on this screened data set.

Table 5 summarizes the results obtained with all methods from five-fold cross validation. (Performance measures in this table are the average of the five-fold cross validation.) We tabulate the number of selected genes (*#* Gene), the number of non-zero coefficients (*#*Para), the highest order of interactions (*q*), and the sum of these three quantities (Total). The individual parameters give different perspectives on the complexity of the model, while the total provides an overall criterion. For prediction accuracy, we calculate the area under the ROC curve, and tabulate this quantity in the column “AUC”. We can observe from these results that pLPS and pLPS3 select fewer genes; pLPS, pLPS3, and Logic Regression use fewer parameters than SPLR; and pLPS and pLPS3 do not go to high order interactions because these are precluded by the model. In the total complexity criterion, there is a tie for first between pLPS and pLPS3. For prediction, as measured by AUC, pLPS is the clear winner.

**Table 5:** Non-Breast Cancer data: Summary of results from five-fold cross validation

**Methods**	**# Gene**	**# Para**	***q***	**Total**	**AUC**
pLPS	9.2	6.6	**2.0**	**17.8**	**0.982**
pLPS3	**8.4**	6.4	3.0	**17.8**	0.945
Logic	14.0	**5.2**	5.0	24.2	0.956
SPLR	17.2	20.6	5.6	43.4	0.962

“Total” sums the number of selected genes, the number of non-zero coefficients in the model, and the highest order of interactions. AUC indicates the area under the ROC curve.

#### Breast cancer survival time

The survival of breast cancer patients depends on many factors, such as grade, stage and oestrogen-receptor status. In this section we study the possible genetic effects using the gene expression barcode data. We denote patients who lived less than 5 years after diagnosis as “cases” and patients who lived more than 10 years after diagnosis as “controls.” Patients with a censored death time less than 10 years and patients that died between 5 and 10 years are excluded. The purpose of this step is not to provide a more homogeneous subset. Rather, we are converting the survival data into a binary outcome, because our method is developed with binary outcomes in mind. After this step, the remaining pool contains 243 patients, among which 80 are cases. The five-year death rate is 80/243 = 32.9*%*.

Formula (11) shows the model fitted by pLPS on this data with 7,654 genes. There are one main effect and four size-two interactions.

(11) $$\begin{array}{l} \;f=3.21-1.59\times \text{PODXL}-2.00\times \text{SYNE}2\;\times \;\text{AKAP}11\\ \;+2.05\times \text{CD}20\times \text{CREB}1-1.88\times \text{STAT}5A\\ \;\times \text{MAPT}-1.89\times \text{MAOB}\times \text{IFFO}1. \end{array}$$

Among the selected genes, CDC20, CREB1, STAT5A and MAPT are known to be related to breast cancer. It was noted in [21] that CDC20 is overexpressed in a large subset of malignancies such as colorectal, breast, lung and bladder cancers. The study [22] reports that CREB1 is much higher in breast tumor tissues as compared to non-neoplastic mammary tissues. Active STAT5 has been identified as a tumor marker of favorable prognosis in human breast cancer, and STAT5 activation is lost during metastatic progression [23]. It has been pointed out by [24] that MAPT inhibits the function of taxanes and high expression of MAPT decreased the sensitivity to taxanes.

As in the previous subsection, we use a screen step to select the most important genes (p-value <10^−3^); this step yielded 592 genes. The cutoff p-value used here is much bigger than that in the non-breast cancer data, because it is small enough to rule out most genes.

Table 6 summarizes the results obtained with all methods from five-fold cross validation. Among the five measures presented, pLPS does best in terms of the highest order of interactions and AUC, winning by a large margin over the other methods in the latter measure. Logic Regression performs surprisingly well in model complexity, selecting the smallest number of genes and parameters. However, its prediction quality, as measured by AUC, suffers from this reliance on overly simple models.

**Table 6:** Breast cancer survival data: Summary of results from five-fold cross validation

**Methods**	**# Gene**	**# Para**	***q***	**Total**	**AUC**
pLPS	10.0	6.8	**2.0**	18.8	**0.824**
pLPS3	10.2	6.6	3.0	19.8	0.780
Logic	**4.4**	**2.6**	3.8	**10.8**	0.721
SPLR	19.4	20.6	5.0	45.0	0.793

“Total” sums the number of selected genes, the number of non-zero coefficients, and the highest order of interactions. AUC indicates the area under the ROC curve.

It is interesting to study the overlap between the sets of genes selected by these different methods. Table 7 shows the results from both data sets (average of the five-fold cross validation). In the breast cancer survival data on the right side of the table, about 40% of the genes are common genes between pLPS and pLPS3. The set of common genes between SPLR and pLPS/pLPS3 contains about 50% of the genes selected by pLPS/pLPS3, and about 25% of the genes selected by SPLR. The absolute number of commons genes between Logic Regression and any others is small, because Logic Regression selected few genes. On the left side of the table, the number of overlapped genes in the non-breast cancer data is relatively small. As previously noted, genes that were used in the comparison stage are all highly correlated with the response in the non-breast cancer data (p-value <10^−8^ compared to <10^−3^ in the breast cancer survival data). It is easier for these methods to replace one gene with another, because they are all of similar importance. Therefore, it is not surprising that the sets of genes selected by different methods do not overlap strongly with each other.

**Table 7:** Summary of common genes

	**Non-Breast Cancer data**				**Breast cancer survival data**			
	**pLPS**	**pLPS3**	**Logic**	**SPLR**	**pLPS**	**pLPS3**	**Logic**	**SPLR**
pLPS	9.2	2.6	1.6	2.0	10.0	4.0	1.0	5.2
pLPS3		8.4	1.6	2.2		10.2	1.0	4.8
Logic			14.0	1.6			4.4	1.6
SPLR				17.2				19.4

Off-diagonal element shows the number of common genes selected by methods from the corresponding row and column. Diagonal element shows the number of genes selected by method from the corresponding row (or column). Numbers are the average of the five-fold cross validation.

## Conclusions

We have described a partitioned version of the LASSO-Patternsearch algorithm (named pLPS) that extends the range of this method to data sets with a higher number of predictors, and allows parallel execution of much of the computation. We show through simulations that pLPS is better than competing methods in selecting the correct variables and patterns while controlling for the number of false patterns in the selected model. By testing on two gene expression data sets, we also show that pLPS gives smaller models with much better prediction accuracy than competing approaches.

Two smoothing parameters with modified tuning criterion are used in pLPS and pLPS3 (in contrast to the single parameter used in LPS). We impose a penalty on the difference between the number of main effects and the number of interactions for pLPS and a penalty on the difference among the numbers of main effects (size-two interactions in pLPS and size-three interactions in pLPS3). These penalties eliminate the extreme cases in which only main effects or interactions arise in the LASSO step, and which the original, unmodified criterion too often produces. On the other hand, if an extreme case is the truth, the LASSO step will generate some false patterns, but the parametric step tends to eliminate them and thus select the correct model.

## Competing interests

The authors declare that they have no competing interests.

## Author’s contributions

WS conceived the method, designed and implemented the pLPS algorithm, analyzed the data and drafted the manuscript. GW supervised the project. RAI provided the data and supervised the project. HCB participated the initial discussion of the method. SJW designed and implemented the algorithm of the minimization problem and participated the writing of the manuscript. All authors reviewed and approved the final manuscript.
